# Clinical progression of patients with COVID-19 in Lagos State, Nigeria

**DOI:** 10.1186/s41231-021-00099-w

**Published:** 2021-09-04

**Authors:** JP. C. Mbagwu, J. O. Olajugba, Paula-Peace James-Okoro, Obidike Blessing

**Affiliations:** 1 Department of Physics, Faculty of Physical Science, Imo State University, Owerri, Nigeria; 2 College of Medicine, University of Lagos, Lagos, Nigeria; 3 Department of Biochemistry, Covenant University, Ota, Ogun Nigeria; 4 Department of Chemistry, Faculty of Physical Science, Imo State University, Owerri, Nigeria

**Keywords:** COVID-19, NCDC, Clinical progression, SARS-CoV-2

## Abstract

**Background:**

The majority of COVID-19 research has been devoted to characterizing the epidemiology and early clinical aspects of the virus. In Lagos, Nigeria, we looked at the temporal progression of COVID-19 patients. We included 1337 confirmed COVID-19 cases in our study from February 27th to March 27th 2020. Of the 1337 patients enrolled, the median age was 50 years old, and 800 (59.83%) were male while 537 (40.16%) were female.

**Method:**

In symptomatic patients, the time from the beginning of signs to admission was 4 (2–7) days. Fever occurred in 217 (16.2%) while cough occurred in 211(15.78%) patients respectively. Patients were given 5–6 treatment, including nutrition support, supplementary oxygen, and antiviral medicines (e.g., Remdesivir, dexamethasone) in a limited percentage of cases. The assessed median period of infection in all patients was 10 days after the start of symptoms (95 confidential intervals [CIs]: 8–11 days). The duration of fever was slightly longer in patients admitted to intensive care units (ICU) than in those who were not (31 days versus 9 days, respectively, *P* < 0.003).

**Results:**

On day 7 after the onset of symptoms, radiological deterioration of the original picture was found in 500 (37.39%) patients. On day 13, 154 of these patients (94.5%) showed signs of radiological improvement. The average time it took for upper respiratory tract samples to test negative for reverse transcriptase PCR was 10 days (90 percent confidence interval: 10–12 days). Virus clearance was more significant in ICU patients than in non-ICU patients (*P* < 0.003).

**Conclusions:**

Community members should continue to adhere to the recommended methods of preventing the spread of COVID-19 infection and patients should seek care early to reduce the risk of mortality associated with the infection as rapidly as possible.

## Background

Dates back towards the end of 2019, cases of an undisclosed emerging disease linked to respiratory tract infection was reported in Wuhan city Hubei province of China [1]. These undisclosed diseases sprang forth and took the lives of many, an estimate of over 2,000 deaths and infection was recorded within the first 10 days of manifestation, few weeks into January 2020, after series of profound analysis on specimens from the lower respiratory tract, a novel virus known as severe acute respiratory syndrome coronavirus 2 (SARS-CoV-2) was reported as the causative agent of the respiratory tract infection [2]. The disease caused by SARS-CoV-2 has been termed coronavirus disease 2019 by a global panel of virus taxonomists (COVID-19) [3]. Delving into history this is not the first time cases of coronavirus has been reported, in 2003 severe acute respiratory syndrome coronavirus (SARs-CoV) was reported in Guadong province of China following the Middle East respiratory syndrome coronavirus (MERS CoV) outbreak reported in South Korea on September 12, 2015 [4].

The Novel Corona Virus Disease SARS-CoV-2 commonly known as the coronavirus disease 2019 (COVID-19) is the third type of coronavirus outbreak as many coronaviruses have been reported from other species. On the 11^th^ of February 2020, an estimate of 114 countries with over 118,000 infected persons having over 4,000 deaths was recorded. Succeeding the widespread and on March 11, 2020, the Director-General of the World Health Organization (WHO) declared the virus a pandemic and enjoined all countries all over the world to take adequate precautionary measures to ensure that the virus is controlled and contained quickly to avoid further spread as there was no safe vaccine at that time [5]. COVID-19 is highly contagious and has a greater tendency of viral transmission to its host receptor when compared to other divisions of the virus, several scientists have established that the coronavirus can be transferred by touching contaminated surfaces [6, 7]. According to this article, the Coronavirus was clear for up to 4 h on a copper surface, 3 h in aerosols, 24 h on a cardboard surface, and 2–4 days on a plastic and stainless-steel surface.

COVID-19 present on the surface of an object does not last long as certain factors like high temperature, the thermal environment may shorten the life span of incubation, although to present, no detailed research has been reported regarding this. Community-based human-to-human transmission through the respiratory droplet, saliva, coughs and sneezing secreted from the nose of an infected person has shown to be the major route by which humans can contact this virus [8–11]. Notwithstanding there is also a potential possibility through Airborne transmission if given great consideration [12, 13]. The commonly reported clinical symptoms of COVID-19 include high fever, dry cough, abdominal pains, respiratory tract infection, severe headache, vomiting, diarrhoea, fatigue, myalgia, sore throat, severe pains, conjunctivitis, loss of taste/smell and difficulty in breathing [14–16].

The virus varies from asymptomatic to fatal which simply means that not every infected person will show symptoms, and a severe acute respiratory infection would not occur in every infected patient. As a result, the virus has three stages: stage I, asymptomatic development with or without visible virus; stage II, non-severe symptomatic period with the virus; and stage III, severe acute respiratory symptomatic phase with high viral dimensions of severe respiratory failure syndrome (SRFS) [17]. On March 20, 2020, the Nigerian government placed travel restrictions on 15 high-incidence countries, including China, Italy, Iran, Norway, South Korea, Spain, Japan, France, Germany, the United Kingdom, the Netherlands, Switzerland, Sweden, and Austria [18], but Nigeria remains one of the 13 top countries liable to high risk of COVID-19 infection. On the 27^th^ of February 2020 Nigeria confirmed her first case of COVID-19 in Lagos state, this virus was brought in by a 44 years old Italian man from Milan who returned to Lagos State on the 25^th^ of February 2020 [19]. Succeeding this affirmation of the index case, within 48 h contact of 216 persons linked to index was traced and followed up immediately of which 45 persons had travel history out of Nigeria, one among the outstanding 176 tested positives to this virus, the virus was managed and controlled by the Nigerian centre for disease control (NCDC) using non-pharmaceutical interventions since there was no safe vaccine to contain the virus [20].

These non-pharmaceutical interventions used by the Nigerian government includes immediate closure of schools and business excluding essential commodities providers such food and drugs, border and restriction on public gatherings, work-from-home arrangements for governmental officials, and airport restrictions, public enlightenment on the constant wearing of a face mask, coughing etiquette, regular washing and sanitizing of hands through SMS, TV and radio stations broadcast, maintaining social and physical distancing of 2 m among humans in public gatherings, effective checking of temperatures in banks and public organizations, implementation of curfew, restrictions on inter-states movements and national lockdowns [21, 22].

The Nigerian government also improved her health care facilities, established emergency operation centres (EOC) liable for screening of travelers, disease surveillance, case management, contact tracing and offering of various laboratory services such as early diagnosis, immediate quarantine and isolation of suspected cases in different states of the federation as well as equipping the NCDC with laboratory diagnostic test kit for COVID-19. As of May 11, 2021 samples of one million nine hundred and seventy-seven thousand four hundred and ninety-seven (1,977,497) out of a population over 205 million was tested but despite all NPI employed several more cases across the states with no travel history out of Nigeria nor related to the contact index has been reported, and Lagos has however remained the epicentre of the pandemic in Nigeria accounting for over one third (35.5%) of the confirmed cases and 16.5% of deaths as of May 11, 2021 [23, 24]. Therefore, assessing the clinical progression of COVID-19 among residence in Lagos is very important.

## Methodology

### Description of study area

Lagos state is situated in the southwestern part of Nigeria. It hosts a population of 14 million people [24], despite being one of the smallest states in terms of landmass by square kilometer in Nigeria. The first recorded case of coronavirus in Nigeria was that of an Italian citizen who returned from Italy to Lagos on the 25^th^ of February 2020 and was confirmed two days later (i.e., 27^th^ of February 2020).

### Study population

The study population are residents of Lagos state.

### Type of study

The study is a retrospective study to determine the clinical progression of COVID-19 among residents of Lagos state. Patient records from isolation centres treating people with mild to severe cases of COVID-19 in Lagos was reviewed.

### Inclusion and exclusion criteria

Only individuals who have been diagnosed as COVID-19 positive via Polymerase Chain Reaction (PCR) or as approved by the Nigeria Centre for Disease Control (NCDC) and are being treated according to the national and World Health Organization guidelines from 27^th^ February to 27^th^ March 2020 are eligible to participate in this study. Individuals showing symptoms of respiratory disease but with negative Polymerase Chain Reaction (PCR) result would be excluded from this study.

### Sample size determination

Sample size was determined by selecting the total number of admissions due to a positive COVID-19 Polymerase Chain Reaction (PCR) within the designated study period (27^th^ February – 27^th^ March 2020). The number of admissions within the study period is one thousand three hundred and thirty-seven (1,337).

### Data collection tool and technique

Data was collected by reviewing the case files of the selected participants with emphasis on the biodata, clinical presentation, laboratory investigations and management instituted according to recommendations from NCDC and WHO.

#### Data management and analysis and presentation

Data was analyzed using the statistical software package, StataCorp 2019.

## Statistical analysis

Continuous variables were reported as percentages, whereas categorical data were reported as means and standard deviations (SD). For comparisons, a two-sided P value of less than 0.05 was considered statistically significant when using the t-test on continuous variables. We show an expressive statistic that was conducted using the statistical software package StataCorp. 2019. Stata statistical software release 16, college station, TX: StataCorp LLC.

### Outcomes

The Lagos COVID-19 pandemic trend and outline, which is located in the southwest of Nigeria and has a population of 14 million people.

**Fig. 1:**
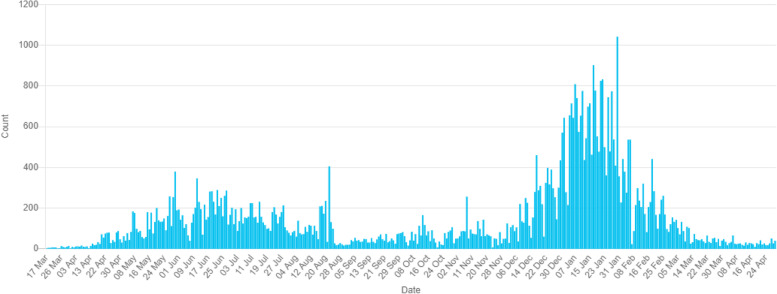
The number of verified COVID-19 cases in Lagos as of April 21, 2021, as a result of more travel and gathering is shown [28]

**Fig. 2:**
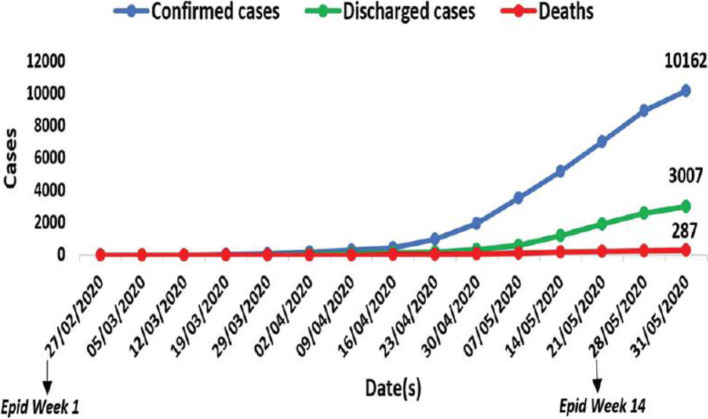
Spread of confirmed Covid-19 cases, discharges, and deaths across Nigeria from 27^th^ February to 31^st^ May 2020 [28]

The first case of the deadly coronavirus in Nigeria was confirmed on February 27, 2020; the case is an Italian citizen who works in Nigeria and returned from Milian, Italy to Lagos, Nigeria on February 25, 2020; nonetheless, this does not indicate the start of an epidemic [25]. Up until April 21^st^ 2021, there were a total of twenty-two thousand five hundred and sixty-two (22,562) cases, twenty-one thousand one hundred and nineteen (21,119) recovered and two hundred and twenty (220) deaths according to official reports, this has been confirmed from Nigeria Centre for Disease Control (NCDC) in Lagos, Nigeria. Figure 1 depicted the epidemical trends of new cases, cumulative cases, and remaining cases, while in Fig. 2 (from 27^th^ February to 31^st^ May 2020) shows the confirmed cases, discharged cases, and deaths, following the first case report, the number of newly confirmed cases per day peaked as a result of more travel and gathering, and the remaining cases began to decline after 13 months (i.e., one year and one month) after April 1st 2021 when there were scarcely any new cases added and the condition improved as of April 21st 2021. We focus on the thousand three hundred and thirty-seven (1337) confirmed patients in the first month to determine the timing and disease progression of COVID-19 (admission date from Feb. 27th to March 27th 2020). Patients were primarily male 800 (59.83 percent) and seen in government-owned COVID-19 treatment facilities. The patients’ average age was 50 years old (IQR, 35 – 63 years) (96 percent) compared to COVID-19 treatment institutions that are privately owned (4 percent). The time from the onset of symptoms to admission to the hospital was 4(2–7) days in patients with symptoms issues related to rigorous care unit’s admission as shown in Table 2. Eighty percent (30.2 percent) of the patients had one, two, or more chronic medical disorders. The lowest daily number sixty-five (65) of COVID-19 confirmed cases were reported and Lagos has forty-one (41) confirmed cases as of 21^st^ April 2021, making it one of the lowest confirmed cases in Lagos, Nigeria. At the time of this paper, many were considered to have mild diseases like cancer, diabetes, and heart which were observed in 402(30.1%), 672(50.3%), and 279(20.9%) of the patients respectively. Only around a quarter of the participants (20.4%) had at least one other condition, and 21.5 percent had several comorbidities. The cases had a mortality rate of 5.4 percent. Fatigue, diarrhoea, and cough which were reported by 49(3.66%), 150(11.11%), and 211(15.78%) of the patients respectively were the most common giving symptoms. Hand cleaning, keeping a physical distance from people (especially those with symptoms), covering coughs and sneezes with a tissue or inner elbow, and keeping filthy hands away from the face are all recommended ways to avoid infection [26, 27].

### Outcomes and treatment of the study population

Negative pressure isolation wards were used for all of these patients. After admission, patients were given supportive care. Patients were given 5–6 treatment, including nutrition support, supplementary oxygen, and antiviral medicines (e.g., Remdesivir, dexamethasone) in a limited percentage of cases. The Lagos management guideline for coronavirus 2019 was used to treat all of the patients (COVID-19). Individualized treatment was not arbitrary and was guided by the severity of the presenting sickness.

## Discussion

The number of infected cases has risen dramatically since the COVID-19 outbreak’s rapid person-to-person transmission in December 2019 [29]. This study used data from NCDC situation reports, which are publicly available on their website, to analyze the pattern and clinical development of COVID-19 symptoms in a cohort of 1337 patients in Lagos, Nigeria.

In this study, the median time from the onset of symptoms to admission was 4 days (IQR 2–7), which was similar to previous findings [30]. Of the 1337 individuals with COVID-19, the majority (99.3%) were symptomatic while only 9 (0.67%) were asymptomatic until admission. The asymptomatic patients can spread the virus to others and become a source of community or hospital-based transmission. However, most of the symptomatic cases had mild symptoms (74.79%) (Table 1). This is consistent with the findings of Abayomi et al. (2021) [32]. The most common clinical presentations, at the onset of illness, in patients with mild symptoms include fever, cough, and fatigue. Fever (16.2 percent) was the most prevalent symptom in patients, which we had similar results to other studies [33, 34]. Dizziness, headaches, and diarrhoea were among the most prevalent symptoms. Some individuals with the infection experienced upper respiratory tract signs and symptoms, such as rhinorrhea (1.42%), shortness of breath (1.12%), or sore throat (1.34%), signifying that the target cells could be in the lower airway, as previously reported [35]. A better therapy is needed to calm the symptoms once they have escalated to a fatal level, which is not beneficial for the patients. Above all, scientists around the world should support the need to develop a good medicine as quickly as feasible for the worldwide benefit of society. Patients that developed chronic symptoms were associated with late presentation, elderly, the lengthier waiting time to hospitalization and medical comorbidities. The following is a list of the comorbidities that have been presented: 237 (70.32%) had no comorbidities, 100 patients (29.67%) presented with comorbidities. Of the 337 patients with severe symptoms, one or more chronic disorders coexisted in 100 of the patients. Cardiovascular and cerebrovascular diseases remained the utmost mutual coexisting illnesses present in 66 patients (4.93%); other underlying conditions included endocrine system diseases in 35 patients (2.61%), digestive system diseases in 11 patients (0.82%), respiratory system diseases in 8 patients (0.59%), chronic hepatitis B virus infection in 4 patients (0.29%) and malignant tumour in 3 patients (0.22%) (Table 1). Similar findings were observed in a study by Wang et al. (2020) [36]. Thus, the predisposing condition for severe COVID-19 symptoms tends to be those with chronic medical conditions. COVID-19 also appeared to be much more prevalent among the men while it spared the women, as 92.7% of the cases were males. Acute respiratory distress syndrome (ARDS) developed in only 16 of the individuals who had severe symptoms. The laboratory analysis results showed decreased lymphocytes, increased C-reactive protein (CRP), increased erythrocyte sedimentation rate (ESR), decreased albumin, increased lactate dehydrogenase, increased lactate and increased CD4 + T cell count (Table 1).

**Table 1:** COVID-19 patients’ clinical presentation and relevant laboratory findings (*N* = 1337) [28, 31]

Symptoms or indications that are clinically significant	
Age, years	
Median (interquartile range)	50(35–63)
Sex	800(59.83%)
Male (N, %)	
Female (N, %)	537(40.16%)
Days from one set of symptoms (days)	4(2–7)
**Admission signs and symptoms (N, %)**	
Fever	217(16.2%)
Cough	211(15.78%)
Fatigue	36(2.69%)
Headache and dizziness	25(1.86%)
Breathing problems	15(1.12%)
Rhinorrhoea	19(1.42%)
Sore throat	18(1.34%)
Diarrhea	10(0.74%)
Lack of appetence	11(0.82%)
Asymptomatic	9(0.67%)
Comorbidity (N, %)	100(7.47%)
Cardiovascular and cerebrovascular diseases	66(4.93%)
Endocrine system diseases	35(2.61%)
Digestive system diseases	11(0.82%)
Respiratory system diseases	8(0.59%)
Chronic hepatitis B virus infection	4(0.29%)
Malignant tumor	3(0.22%)
**Laboratory findings**	
White blood cells (× 10 ^9^ per L)	4.71(3.80–5.86)
Lymphocytes (× 10 ^9^ per L)	1.12(0.79–1.49)
C-reactive protein (mg/L)	12(4.4–29.4)
Erythrocyte sedimentation rate (mm/h)	54(33–90)
Alanine aminotransferase (U/L)	23(15–33)
Aspartate aminotransferase (U/L)	25(20–33)
Albumin (g/L)	40.8(37.9–43.0)
Lactate dehydrogenase (U/L)	229(195–291)
Lactate (mmol/L)	1.4(1.1–2.1)
Estimated glomerular filtration rate (mL/min/1.73 m^2^)	109.2(95.3–127.2)
CD4 T cells counts (cells/uL)	431(299–637)
CD4/CD8 ratio	1.68(1.241.33)

The decrease in albumin suggested that nutritional supplementation was necessary. The reduced lymphocyte and CD4 + T cell count indicates that COVID-19 inhibits immune cell function by consuming the cells. ESR and CRP elevations are linked to an inflammatory response and cytokine storm triggered by viral infection. These results correlate with other studies [37]. The destruction of T lymphocytes and cytokine storms may indicate a more significant risk for aggravation of symptoms in patients (Table 2). This could also be used as a reference index for diagnosing SARS-CoV-2 infection. All the patients underwent antiviral therapy and this was beneficial to the treatment of the infection. Because antibiotic therapy was strictly monitored and only given to individuals with a highly suspected bacterial disease, it was only provided to a small percentage of patients (8.15%). In severe cases, corticosteroids and gamma globulin were given to decrease the inflammatory reaction in the lungs. Along these lines, in addition to powerful antiviral medicines that can reduce viral load at an early stage, resulting in less immunopathological damage, host-directed therapy could be a useful tool in lowering mortality. Mechanical ventilation was used as the main supportive treatment for severe patients who developed ARDS. In total, 29 people died, the majority of them were elderly and/or had coexisting medical conditions (Table 3). At the end of the study's deadline, (March 27th, 2020), 188 patients showed improvements and were discharged, 38 patients continued to be hospitalized while 19 were moved to the intensive care unit (ICU) as they did not improve significantly after viral control and the latter developed other complications like a respiratory failure. This shows that there was a high recovery rate among the patients. An effective antiviral to reduce the viral load early may be beneficial to reduce the risk of disease progression and poor outcome. Several medicines, including remdesivir, are being tested in clinical trials as possible inhibitors of SARS-CoV-2 replication. Some severe patients required high-flow nasal cannula (8.67%), non-invasive ventilation (2.09%), invasive mechanical ventilation (1.12%) and BiPAP (3.66%) (Table 4). As a result, old age and the existence of comorbidity may be linked to an increased risk of death. In conclusion, the majority of COVID-19 infections are minor. The two-phase pattern of illness progression shows that early viral replication reduction and later use of host-directed therapy are required to improve CVOID-19 prognosis.

**Table 2:** Issues related to rigorous care unit’s admission [31]

	Univariate logistic regression			Multivariate logistic regression		
	OR	97% CI	*P* value	OR	97% CI	*P* value
Age	3.10	3.04–1.13	< 0.003	**3.06**	**3.00–1.22**	**0.068**
Male	9.10	4.04–24.07	0.004	5.38	2.77–14.19	0.33
Comorbidity	6.34	3.70–11.10	0.004	3.83	2.50–6.95	0.58
Fever	3.52	2.34–6.85	0.26			
White blood cells (× 109 per L) Lymphocytes (× 109 per L) C-reactive protein (mg/L)	3.28 2.24 3.04	3.08–1.52 2.08–0.75 3.02–1.05	0.006 0.03 < 0.003	3.07 6.05 3.01	2.79–1.65 2.89–18.25 2.99–1.23	0.88 0.28 0.72
Erythrocyte sedimentation rate (mm/h)	3.00	2.99–1.02	0.78			
Albumin (g/L)	2.75	2.66–0.85	< 0.001	2.95	2.78–1.26	0.82
Alanine aminotransferase (U/L)	3.01	3.0–1.03	0.16			
Aspartate aminotransferase (U/L)	3.02	3.0–1.03	0.06	2.99	0.96–1.03	0.97
Albumin (g/L)	2.75	2.66–0.85	< 0.003	2.95	0.78–1.16	0.80
Lactate dehydrogenase (U/L)	3.01	3.0–1.02	< 0.003	3.01	1.0–1.02	0.28
Lactate (mmol/L)	3.23	2.88–1.70	0.33			
Estimated glomerular filtration rate (mL/min/1.73m2)	2.98	2.96–0.99	< 0.003	1.02	0.99–1.02	0.99
CD4 T cell counts (Per 100 cells/uL)	2.45	24.31–0.64	< 0.001	**0.77**	**0.55–0.94**	**0.04**
Radiological lesion	6.46	4.62–31.9	0.16

**Table 3:** Management of COVID-19 patients in the clinic [38]

The period from admission to discharge	All patients (*N* = 1337)
**Definitions of severe patients**	
Respiratory rate > 35/min	212 (15.85%)
Mean oxygen saturation < 93%	195 (14.58%)
PaO_2_/FiO_2_ ≤ 300 mmHg	89 (6.65%)
Lung lesion progression > 50% within 24–48 h	26 (1.94%)
**Treatment**	
Antiviral therapy	276 (20.64%)
Antibiotic therapy	266 (19.89%)
Use of corticosteroid	109 (8.15%)
**Oxygen support**	
Nasal catheter	186 (13.91%)
Mask oxygen	89 (6.65%)
High-flow nasal cannula	116 (8.67%)
**Assisting ventilation**	
Bipap	49 (3.66%)
Invasive mechanical ventilation	15 (1.12%)
**Outcome**	
Discharge	188 (14.06%)
Continue to be hospitalized	38 (2.84%)
ICU	19 (1.42%)
Death	29 (2.16%)

**Table 4:** The treatment of patients who are progressing and those who are stable [39]

	**Total (*N* = 1337)**	**Mild‑moderate (*N* = 1000)**	**Severe‑critical (*N* = 337)**	***P* value**
Treatment	1337	1000	337	
Antiviral therapy				
Antibiotic therapy	109 (8.15%)	61 (4.56%)	58 (4.33%)	< 0.002
Use of corticosteroid	99 (7.40%)	32 (2.39%)	78 (5.83%)	< 0.002
Use of gamma globulin	91 (6.80%)	28 (2.09%)	73 (5.54%)	< 0.002
Regulate intestinal flora	189 (14.13%)	133 (9.94%)	66 (4.93%)	< 0.002
Oxygen support	248 (18.54%)	170 (12.71%)	89 (6.65%)	
Nasal cannula	191 (14.28%)	167 (12.49%)	33 (2.46%)	< 0.002
Mask oxygen inhalation	9 (0.67%)	5 (0.37%)	4 (0.29%)	0.047
High-flow nasal cannula	12 (0.89%)	0	10 (0.74%)	
Non-invasive ventilation	26 (1.94%)	0	28 (2.09%)	
Invasive mechanical ventilation	13 (0.97%)	0	16 (1.19%)	
Invasive mechanical ventilation + ECOM	7 (0.52%)	0	7 (0.52%)	
IL-6 (pg/mL)	10(0.74%)	4(0.4%)	6(1.7%)	< 0.001
Acute respiratory distress syndrome	16 (1.19%)	0	16 (1.19%)	

## Conclusion

Finally, this research adds to the growing body of knowledge about the onset and course of clinical symptoms in COVID-19 patients in Lagos, Nigeria. Respiratory symptoms and fever are the most common symptoms in infected patients, especially in the early stages. The cure and recovery rate of patients with COVID-19 is quite high. Late presentation, longer waits to hospitalization, advanced age and coexisting chronic illnesses brought about severe symptoms which could be an analyst of death. Males, patients with comorbidities and advanced age had subordinate COVID-19 results, higher risks and occurrences; thus, emphasis should be placed on them. These variables, along with a decrease in T cells and a high level of CRP, can aid in the early detection of COVID-19 clinical development. Community members should continue to adhere to the recommended methods of preventing the spread of SARS-CoV-2 infection and to lower the risk of infection-related death, patients should seek treatment as soon as possible.

## Data Availability

Not Applicable.

## Abbreviations

NCDC: Nigeria Center for Disease Control
WHO: World Health Organization
CRP: C-reactive protein
PCR: Polymerase Chain Reaction
